# Using Genomic Sequence Information to Increase Conservation and Sustainable Use of Crop Diversity and Benefit-Sharing

**DOI:** 10.1089/bio.2018.0043

**Published:** 2018-10-12

**Authors:** Michael Halewood, Isabel Lopez Noriega, Dave Ellis, Carolina Roa, Mathieu Rouard, Ruaraidh Sackville Hamilton

**Affiliations:** ^1^ Bioversity International, Rome, Italy.; ^2^ International Potato Center, Lima, Peru.; ^3^ Centro Internacional de Agricultura Tropical, Cali, Colombia.; ^4^ Bioversity International, Montpellier, France.; ^5^ International Rice Research Institute, Los Baños, Philippines.

**Keywords:** genomic sequence information, conservation, sustainable use, benefit-sharing

## Abstract

This article describes how CGIAR centers and partners are using genomic sequence information to promote the conservation and sustainable use of crop genetic diversity, and to generate and share benefits derived from those uses. The article highlights combined institutional, and benefit-sharing-related challenges that need to be addressed to support expanded use of digital sequence information in agricultural research and development.

## Introduction

The DNA of every living organism on earth encodes the basic building blocks of its life. DNA sequences are unique from one organism to the next; they can aid in taxonomic classification, identification of unique genes, and gene combinations that encode valuable traits for sustainable agriculture under changing climates. While whole-genome sequences are increasingly available as a result of new-generation technologies,^1,2^ our collective capacity to actually analyze and benefit from that data is lagging behind.^3^ Currently, genetic markers—minuscule segments of DNA scattered throughout the whole genome—are successfully deployed to genotype individuals and, with some success, to identify individuals in breeding programs that contain traits of agronomic importance. Genotyping is a powerful tool to help identify gaps in collections of plant genetic resources for food and agriculture (PGRFA).^4,5^ Unique genetic fingerprints of existing crop varieties can provide a baseline to assess losses of farmers' varieties, concomitant priorities for *in situ* conservation, and reintroduction into local production systems of “lost” farmers' varieties that have been conserved *ex situ*.

Plant varieties (including farmers' varieties, bred varieties, and wild ancestors) represent combinations of genetic sequences that underpin the traits of each particular variety in interaction with the environment where the variety grows. While a variety may be unique as a whole, individual genetic sequences (e.g., coding an early-maturing or late-maturing trait) may be expressed in the same manner in many different varieties. The reverse can also occur: the same trait, for example, early-maturing, can also be under the control of different sequences in different varieties. The expression of any given gene can be widely influenced by environment. The uniqueness of a variety comes from the combination of those genetic sequences and the environment's influence. The combination in any particular variety is the result of hundreds and thousands of years of random, environmental, farmer, and breeder selection.

While a powerful tool, genetic sequence data cannot be used in isolation from other technologies, including phenotyping (i.e., the study of the observable traits of organisms). While genome sequencing and genetic fingerprinting may help to distinguish “what is the same” and “what is different” genetically, morphological (phenotypic) data are needed to fully interpret the digital sequence. Most traits, particularly those related to abiotic stresses, are under complex genetic control involving multiple forms of multiple genes interacting in networks.^6–8^ A crop's ability to tolerate drought, for example, depends on the anatomy and architecture of roots, leaves and stems; the rate of progress through the life cycle in relation to the development of drought; and difficult-to-measure attributes of photosynthetic, respiratory, and other biochemical and physiological capacities of the plant.

This article describes how CGIAR centers and partners are using genomic sequence information to conserve and sustainably use crop genetic diversity, and to generate and share benefits associated with those uses. The article also includes reflections from CGIAR centers concerning technical and policy challenges that need to be addressed for the enhanced use of genomic sequence information in the future.^9,10^

CGIAR's mission is to advance agricultural science and innovation to enable poor people to better nourish their families, and improve productivity and resilience so they can share economic growth and manage natural resources in the face of climatic changes and other challenges. Fifteen CGIAR centers carry out research for development under this mission in close collaboration with hundreds of partners, including national and regional research institutes, civil society organizations, academia, development organizations, and the private sector. Most of CGIAR's (CGIAR, formerly the Consultative Group for International Agricultural Research) work is focused on developing countries and regions. Eleven CGIAR centers host international PGRFA collections under the framework of the International Treaty on Plant Genetic Resources for Food and Agriculture (ITPGRFA). These centers use genomic sequence information to improve their conservation and sustainable use of crop genetic diversity. Some CGIAR centers also generate and use fish, livestock, and microbial genomic data. CGIAR centers are involved, either as leaders or members of research consortia, in whole- or high-density genome sequencing and genotyping of a number of crop plants, including banana,^11^ cassava,^12^ chickpea,^13^ cowpea, groundnut,^14^ millets,^15^ maize, pigeon-pea,^16^ potato,^17^ rice,^18^ sorghum, sweetpotato, wheat, and yam.^19^

## Using Genomic Sequence Information for Crop Diversity Conservation

Managers of *ex situ* collections have long sought to define the diversity of crops they hold, to know better what they are conserving and to identify gaps in their collections. Morphological descriptors have been developed and used to characterize *ex situ* collections, but these descriptors have limited value in assessing closely related individuals and in defining the range of genetic diversity that exists within and between landraces. Characterization of diversity found *in situ* remains an elusive goal. However, it will be achievable when whole *ex situ* collections and representative samples of *in situ* diversity can be sequenced.^20^ DNA sequence data, once obtained on a sufficiently large scale, will be the best tool for describing and analyzing the extent of diversity *in situ* and to aid long-term conservation of maximum diversity so that all elements of the diversity present in any one crop are conserved long-term for humankind.

**FIG. 1.**
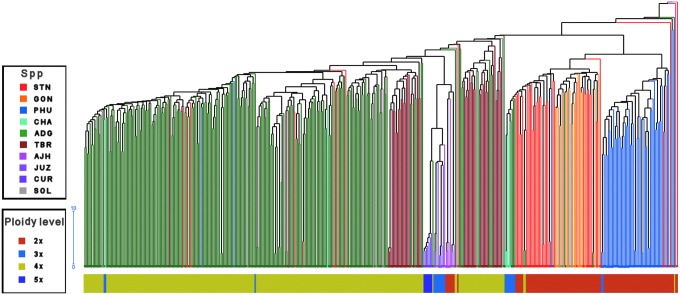
Dendogram of the CIP diversity reference core set of landrace cultivated potato (*Papa nativa*) based on genetic data from the SolCAP 12K SNP array. *Colored lines* in the dendogram denote different potato species. *Bar* below the dendogram denotes ploidy level (CIP data, unpublished). CIP, International Potato Center; SNP, single nucleotide polymorphism.

Since the 1980s, multiple molecular (DNA) markers and associated techniques have been developed. Some of them, suitable for research on population diversity, evolution, gene flow, and inheritance of traits, are increasingly used to better understand the diversity conserved in international collections and facilitate researchers and breeders' work by identifying potential sources of useful traits (Table 1). CGIAR has embraced these new technologies to better characterize crop diversity, and understand the relationships among conserved accessions. Furthermore, CGIAR takes advantage of these technologies to identify gaps in the international “in trust” PGRFA collections they host, identify materials for collection, and identify materials for reintroduction into *in situ* conservation and use programs.^4,21,22^ For example, the International Potato Center (CIP) recently genotyped their entire *ex situ* collection of cultivated landrace potato and sweetpotato, laying the foundation for assessing the diversity present in such collection (Fig. 1).^23^ Among other things, this information has been useful for identifying what materials can/should be restored to Peruvian indigenous communities. In Peru, farmers traditionally cultivated 20–40 different landraces of potatoes as a form of insurance; by planting such diversity, some landraces will produce a crop even in bad years, sustaining farmers until the following harvest. Over the past several decades, the planting of many different potato landrace varieties by some communities has gradually decreased and many families now plant fewer than 10 varieties. One challenge associated with potato restoration work is that CIP scientists and local farmers do not know what diversity existed 50 or 100 years ago … or even what diversity exists today in the *ex situ* gene bank. Genotyping the international potato collection hosted by CIP has helped to reveal the relatedness of individuals within the collection and can be used to identify novel alleles that are not conserved *ex situ* when similar genotyping is done *in situ*.

**Table 1.** Molecular Markers for Diversity Characterization and Breeding Applications

*Molecular marker*	*Basis of polymorphism*	*Level of polymorphism*	*Suitable for*	*Advantages*	*Disadvantages*
RFLPs	Different sizes of alleles associated with restriction fragments generated by enzymes (endonucleases)	Medium	Genetics, e.g., to find where a specific gene lies on a chromosome; gene flow; phylogenetic studies	First applied DNA marker for genotyping; useful in construction of genetic linkage maps	Requires *a priori* knowledge of studied DNA sequence and a large sample size; technically and time-wise high demanding; difficult to automate; limited coverage of genome (low copy coding region); rarely used now
RAPDs	Different sizes of alleles based on length of short primers complementary to randomly targeted DNA in multiple locations	High	Diversity, e.g., closely related species; gene mapping	Cheap; technically and time-wise low demanding; produces large no. of bands that can be characterized individually	Low reproducibility; mainly dominant; difficult to analyze; difficult to automate; cross-study comparisons are difficult
AFLPs	Differences in length of selectively amplified restriction fragments generated by endonucleases	High	Diversity and genetics, e.g., population structure studies; evaluation and characterization of animal and plant resources	Large numbers of markers can be generated	Low reproducibility
SSRs	Simple sequence repeats in tandem from 1–6 nucleotides in length	High	Diversity, genetics, and breeding, e.g., to distinguish closely related genotypes (population studies); linkage disequilibrium studies (i.e., association of a disease-causing locus and a marker)	Highly informative (large no. of alleles, high heterozygosity); codominant; easy to isolate; low ascertainment bias	High mutation rate; complex mutation behavior; not abundant enough; difficult to automate; cross-study comparisons require special preparation
SNPs	Single nucleotide mutation at a specific place (locus) in a DNA sequence	High	Diversity, genetics, and breeding, e.g., genetic variation in different species and breeds	Low mutation rate; high abundance; easy to type; high potential for automation; cross-study comparisons easy	Substantial heterogeneity rate among sites; expensive to isolate; low information content of a single SNP
GBS	Sequences of the ends of all resulting DNA restriction fragments produced by a frequent cutter enzyme; generates large no. of SNPs	High	Genetic map construction; SNP genotyping in a variety of species and populations useful for breeding, plant genetics, and germplasm characterization	Useful for high diversity and large genome species; cost-effective for genomic-assisted breeding; high automation; technically easier to use and less demanding than RADseq	Management and analysis of large amount of data; proprietary technology
DArTseq	Works on a genome complexity reduction concept—selection of genome with predominantly active genes (target low copy sequences)	High	High-resolution mapping and detailed genetic dissection of traits; phylogeography (in animals); genetic relatedness of species; species origin studies	Reduction complexity methods are simple and cheaper than other GBS-based methods; high reproducibility; high heterozygotes representation	Management and analysis of large amount of data; single source for proprietary technology
RADseq	Sequences of short regions (50–150 bases) flanking each and all restriction sites for a given endonuclease	High (uncovers 100 s–1000 s polymorphic genetic markers in a subset of genome)	Population differentiation and selection studies; phylogeography; ecological and evolutionary genomics; linkage mapping	Relatively low cost (greater no. of samples) and simple; greater coverage per locus; no prior genomic information required	Bias due to allele dropout, PCR duplicates, and variance in depth of coverage among loci (all of the former vary according the RADseq method used)

AFLP, amplified fragment length polymorphism; DArTseq, diversity arrays technology sequencing; GBS, genotyping by sequencing; RADseq, restriction site-associated DNA sequencing; RAPD, randomly amplified polymorphic DNA; RFLP, restriction fragment length polymorphism; SNP, single nucleotide polymorphism; SSR, simple sequence repeat.

**FIG. 2.**
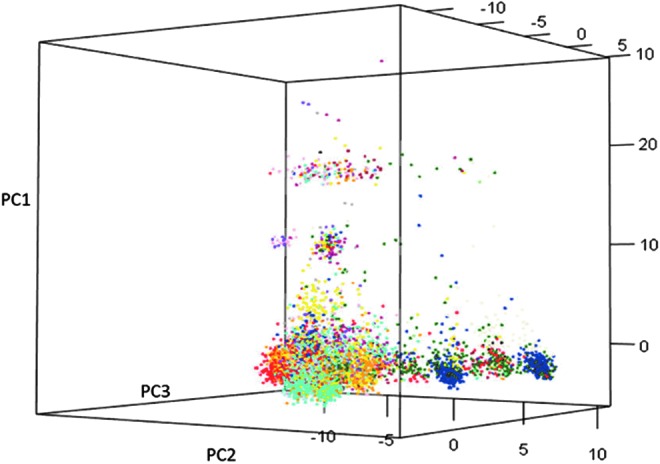
Three-dimensional PCA graph showing the distribution of Mexican wheat landrace groups based on genetic markers. There were a total of 15 groups—each represented by a different color—that correspond to different Mexican states. PC1, PC2, and PC3 contribute 10.5%, 8.2% and 6.9% of the total variation, respectively. Source: Vikram et al.^25^ PCA, principal component analysis.

It is beyond the capacity of plant breeders and other users to sample and use all the genetic diversity of particular crops contained in the CGIAR collections. To help users find what they need, CGIAR centers' gene banks develop smaller, core sets that represent most of the diversity in the overall collection, or portions of the collection.^24^ For example, CIMMYT (International Maize and Wheat Improvement Center, CIMMYT is a Spanish acronym) recently developed a core set representing the diversity of Mexican wheat landraces. Introduced into the Americas between the 16th and 18th centuries, wheat became gradually adapted to local environments, giving rise to Mexican wheat landraces known as “creole wheats.” As such, they should have useful genetic variation for heat and drought stress tolerance. Scientists from CIMMYT and Mexico's Instituto Nacional de Investigaciones Forestales, Agrícolas y Pecuarias (INIFAP) and the Punjab Biodiversity Board (India) carried out a study to: (1) characterize the collection of Mexican wheat landraces conserved at the CIMMYT gene bank; and (2) develop a core set using multiple variables. Eight thousand four hundred sixteen wheat landraces representing a range of Mexican agro-ecologies were characterized using genetic markers (DArTseq) and phenotypically for yield potential, drought and heat tolerance, and yellow rust resistance to identify a core set capturing 89% of the rare alleles present in the complete set (Fig. 2).^25^

Genomic sequence information is very useful in creating these core sets, ensuring representation of collections' genetic diversity in the core set. Genomic sequence information also provides guidance for breeders to make use of these core sets in effective, efficient ways. Some CGIAR centers, in collaboration with local and national organizations, have applied genomic tools to genetically characterize landrace diversity found *in situ* with the aim to promote conservation of landraces and traditional varieties through their cultivation and commercialization by farmers. For instance, the Heirloom Rice Project, coordinated by the Philippine Department of Agriculture and the International Rice Research Institute (IRRI), has genotyped 124 traditional varieties of rice to show how related each variety is to others from the same location or from other provinces. This work will increase farmers' knowledge of their heirloom rice diversity and their market opportunities.^26^

## Using Genomic Sequence Information for Sustainable Use

Conventional plant breeding relies on natural or induced genetic variation combined with efficient selection of favorable genetic combinations and evaluation of phenotypes to identify variants of interest for desirable traits. The use of genomic information can shorten these processes and make them more precise. The use of molecular markers (Table 1) has allowed CGIAR scientists to identify genes that control important traits. For example, IRRI researchers identified a gene that enhances rice germination under anaerobic conditions. Tolerance of anaerobic soil during germination enables uniform germination and seedling establishment under submergence, and is a key trait for the development of tropical direct-seeded rice.^27^ Researchers from the International Crops Research Institute for the Semi-Arid Tropics (ICRISAT) have identified the molecular markers for quantitative trait loci (QTL) influencing grain iron and zinc content in sorghum, with the long-range potential impact of reducing malnutrition in sorghum-producing and -consuming countries.^28^ Researchers from the Centro Internacional de Agricultura Tropical (CIAT) and the International Institute of Tropical Agriculture (IITA) have identified QTL associated with resistance to cassava green mite, cassava mosaic disease, cassava brown streak disease, high provitamin A, and high dry matter content in storage roots.^29,30^ CIMMYT researchers have developed and deployed breeder-ready production markers for provitamin A content, maize streak virus resistance, maize lethal necrosis (MLN) resistance, and high haploid induction rate.

Digital genetic sequence data obtained from high-throughput genotyping techniques have dramatically enhanced CGIAR centers' ability to screen highly diverse germplasm from international collections at a large scale to identify allelic variation linked to agronomic traits. Genome-wide association studies conducted by CGIAR researchers for rice,^31,32^ maize,^33,34^ peanut,^35^ sorghum,^36^ banana,^37^ and cassava^38,39^ have led to the identification of useful diversity and molecular markers for a wide range of traits. For example, ICRISAT identified genetic markers associated with 36 agronomically important traits in peanut gene bank accessions, opening new prospects for future breeding programs.^35^

CGIAR centers are also involved in using genetic information to conserve and enhance underutilized plant species. The World Agroforestry Centre (ICRAF) is a member of the African Orphan Crops Consortium (AOCC), which seeks to improve the livelihoods of smallholder farmers by improving 101 orphan crops, including trees, and strengthening associated value chains. As part of this work, AOCC is sequencing whole genomes and developing single nucleotide polymorphism (SNP) panels. Through the African Plant Breeding Academy, AOCC is training ∼120 African plant breeders in national agricultural research programs to use genomics tools to take advantage of the genomic sequence data that is being generated.

CGIAR centers and partners are also using genetic sequence information to make useful contributions to farmer-led improvement programs, as a means of completing farmers' knowledge and adding to the knowledge base for local-level management and future enhancement efforts. For example, Bioversity International's Seeds for Needs programme uses a combination of participatory approaches, genomics, and quantitative genetics to trace the genetic basis of smallholder farmer preferences of durum wheat traits in Ethiopia. Communities evaluated 400 Ethiopian wheat varieties, mostly landraces, for traits of local interest in two locations in the Ethiopian highlands. For each wheat variety, farmers provided quantitative evaluations of their preference for flowering time, spike morphology, tillering capacity, and overall quality. Ten agronomic and phenology traits were simultaneously measured on the same varieties, providing the means to compare them with farmer traits. The durum wheat varieties were genotyped for more than 80,000 SNP markers, and the resulting data were used in a genome-wide association study that resulted in a molecular dissection of smallholder farmers' choice criteria. One hundred twenty-four putative QTL affecting farmer-preferred traits and 30 putative QTL affecting metric traits were found. The study showed that smallholder farmers' traditional knowledge can be associated with QTL for desired phenotypes. The combination of participatory variety selection and modern plant breeding can speed up the genetic gain in breeding that targets smallholder farming systems and also lead to improved varieties more closely addressing smallholder farmers' needs.^40^

### Genome engineering and synthetic biology

Since the 1970s, the focus of so-called “genetic modification” has been to target the insertion of new genetic material into the genomes of organisms and to control their expression. A distinct new generation of technologies, commonly known as genomic editing, is now available to induce mutations at preselected loci to disrupt the function of one or more specific genes; to alter existing sequences to reproduce ancient alleles or to introduce novel alleles; and to introduce new genetic material into specific loci or regions of the genome. Genome editing could vastly reduce the time to create new varieties by facilitating the direct modification of (un)favorable alleles in germplasm, thus reducing the number of breeding cycles required. Equally importantly, genome editing now offers a fast and economical method to validate the function of native genes, thus speeding up conventional marker-assisted breeding.

CIMMYT is using the CRISPR/Cas9 to edit genes for stress tolerance and quality traits in maize and MLN, a disease prevalent in East Africa that poses a significant threat to food security in that region. CIMMYT will gene-edit wheat for durable rust resistance and improved metal ions (zinc and iron). CIAT started genome-editing research in 2014 with rice, and has since expanded its work to cassava and beans. CIAT's work on rice focuses on developing resistance to viruses and bacteria, improving nutritional quality and attaining hybrid seeds; its work on cassava seeks to improve starch quality and generate resistance to herbicides. The objective of CIAT's work on beans is to increase nutritional quality and early detection of pathogens for diagnostic purposes. Other CGIAR centers are also include gene-editing techniques as part of their crop improvement programs.

### Genomic information systems for sustainable use of plant genetic resources

**FIG. 3.**
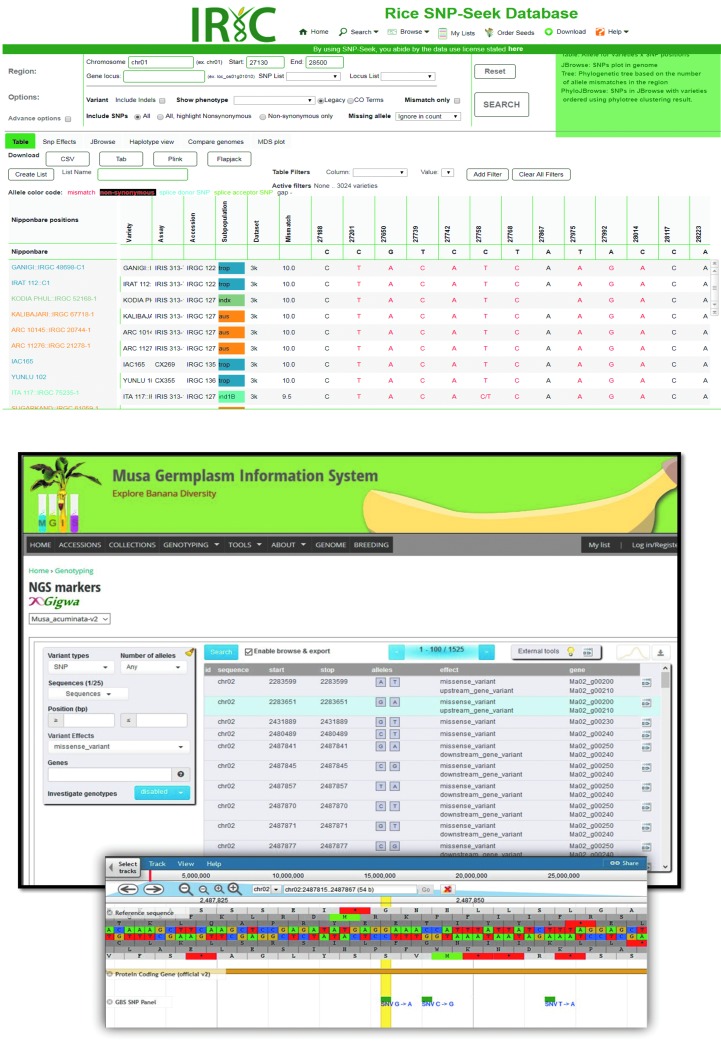
Overview of the SNP-Seek Database^d^ that enables queries concerning 3024 sequenced rice accessions, and the MGIS information system^e^ that facilitates exploration of allelic gene diversity in a panel of banana (*Musa spp.*) accessions.

Extensive genotypic data, linked to measured traits, allow germplasm repositories to be searched for materials containing desired genetic elements or trait characteristics for direct use in production or in breeding programs. CGIAR centers have been involved in developing information systems for a number of crops, including the Rice SNP-Seek Database^41^ and the Musa Germplasm Information System^42^ (Fig. 3).

CGIAR centers share information on genetic resources held in trust through their own databases and through the global online gateway Genesys.^a^ CGIAR is participating in the creation of the Global Information System on PGRFA (GLIS^b^) under the framework of the ITPGRFA. CGIAR gene banks have started to issue digital object identifiers (DOI) for all genetic resources they maintain in their international PGRFA collections. These DOIs will provide a means of ensuring that research results, including genomic research, are associated with and traceable to the germplasm held in trust.

## Using Genomic Sequence Information to Generate and Share Benefits

In recent years, there has been a surge in interest, on the part of intergovernmental bodies dealing with the conservation and use of biological diversity, concerning the governance of genomic sequence information. The conference of the parties of the Convention on Biological Diversity (CBD) and the meeting of the parties of its Nagoya Protocol, the governing body of the ITPGRFA, and the FAO Commission on Genetic Resources for Food and Agriculture have all launched processes to gather information about how genomic sequence data are used, to set the stage for future discussions of governance options.^c^ A growing number of developing countries are urging these bodies to require benefit-sharing from commercial users of genomic sequence data. CGIAR has made submissions to these bodies concerning CGIAR centers' uses of genomic sequence information.^43^

### Nonmonetary benefit-sharing

Improved plant varieties—including the increasing proportion of those whose development depends in some way on the use of genomic sequence data—are used by farmers the world over, generating household income and food security; at a larger scale, they contribute to national and regional economic development. Under the CBD, Nagoya Protocol, and ITPGRFA, income generated by farmers from the use of improved resources and local, national, and regional economic development are considered nonmonetary benefits. Monetary benefit-sharing is more narrowly construed to refer to payments from organizations who commercialize new genetic resource products to the providers of the genetic resources from which the products are derived.

CGIAR pursues its mission by working through partnerships to create nonmonetary benefits for farmers and national programs in developing countries. Perhaps the most important overall benefit from CGIAR's use of genomic sequence information is “food and livelihood security benefits” (one of the nonmonetary benefits listed in the Annex to the Nagoya Protocol). In the previous sections, we have provided examples where genomic information played a critically important role in the discovery of genes that contributes to the development of crop varieties that are more productive, nutritious, disease-resistant, less reliant on chemical inputs, and adapted to changing climatic conditions. Given the relatively recent emergence of next-generation sequencing technologies, there are still not many examples of fully developed research and development chains that start with the generation of raw sequence data and end with released cultivars and breeds adopted by farmers. However, it is clear that use and reliance of genomics data in crop improvement programs will increase over time.

One challenge related to the evolution of new genomic-sequencing technologies will be to ensure they are used for sustainable development, generating benefits for resource-poor farmers in developing countries. There is a risk that new technologies/methods may disproportionately benefit developed countries, and perpetuate rather than diminish the existing North/South technology divide. To ensure next-generation sequencing technologies benefit the Global South, national agricultural research organizations of developing countries must be engaged as equal partners in identifying challenges to be addressed through genomics-based research.^44^ They must also be partners in the actual conservation, research, and development work.^10^ In the following paragraphs we provide examples of how CGIAR centers partner with, train, and transfer technology to agricultural research organizations in developing countries.

#### Addressing climate change challenges through co-generation and transfer of technologies for genetic characterization and crop improvement

CIMMYT, Bioversity, International Centre for Agricultural Research in the Dry Areas (ICARDA), and IRRI have partnered with national research organizations from 13 countries in Africa and South Asia to co-generate and share technologies for genetic characterization and marker-assisted improvement of wheat, barley, and rice, focusing on traits and alleles that are important for the crops' adaptation to climatic changes. These efforts are taking place under four projects funded by the ITPGRFA Benefit Sharing Fund (BSF). Training of plant researchers, breeders, and informaticians from the countries involved and from other target countries on the use of genomic tools is a central part of the four projects.^45^

#### Increasing African breeding programs' capacities to incorporate genomic tools in sweetpotato improvement

CIP is a partner in the Genomic Tools for Sweetpotato Improvement Project that aims to develop modern genomic, genetic, and bioinformatics tools to facilitate crop improvement and improve genetic gains in sweetpotato. Sweetpotato is an important food security and cash crop with potential to alleviate hunger, vitamin A deficiency, and poverty in Sub-Saharan Africa. It is mainly grown by poor women farmers in small plots. The project supports capacity development. Traditional and web-based training workshops and seminars are organized for partner organizations in Africa to facilitate access to and use of molecular markers in breeding (https://sweetpotatogenomics.cals.ncsu.edu).^f^

#### Tools, training, and joint research using maize and wheat sequence information in Latin America

The CIMMYT-led “MasAgro Biodiversidad” promotes use of wheat genetic resources through the creation of a platform linking germplasm, data, software tools and services, and capacity-building. It has developed free distance-learning modules focusing on (1) the theory of genotypic data, and technologies to generate and analyze it, and (2) practical use of an Android application to record phenotypic data of maize and wheat varieties in the field^g^. In 2016 and 2017, MasAgro Biodiversidad supported 13 projects led by Mexican scientists/organizations focusing on characterization of yellow rust resistance of Mexican wheat accessions, genomic characterization of maize in University of Guadalajara gene bank, informatics models to select gene bank accessions based on allele frequency, genetic resources for forage maize in highland Mexico, mapping traits associated with highland adaptation in Central Mexico, nutritional quality of maize, identification of molecular markers associated with expansion (popping quality) in popcorn maize, and searching for sources of resistance to Karnal bunt disease in wheat. Forty-two students have pursued PhD, MSc, or BSc thesis projects within MasAgro Biodiversidad (Kevin Pixley, pers. comm.).

Shared research results based on genetic resources are another critically important benefit. CGIAR's default policy is to make all research results, including data, freely available, as set out in the CGIAR Principles for Management of Intellectual Assets in 2012 and open access policy in 2013. The Intellectual Assets Principles allow CGIAR centers to restrict access to an intellectual asset if necessary for the further development or dissemination of that asset in furtherance of the CGIAR mission. However, even where such restrictions are justified, the policy specifies that the asset must be freely available to public organizations in developing countries for research and breeding.^46,47^

### Monetary benefit-sharing

The ITPGRFA establishes the monetary benefit-sharing rules that apply to most of the CGIAR centers' research and development work that involved accessing, conserving, improving, and distributing PGRFA. By extension, recipients of PGRFA from CGIAR centers are also bound by the terms of the ITPGRFA's multilateral system of access and benefit-sharing. Accordingly, commercializers of new PGRFA products derived from germplasm they receive from CGIAR centers are obliged to pay 0.77% of sales to the ITPGRFA's BSF if those products are not made available for use by others for further research or breeding.

The ITPGRFA (like the CBD and the Nagoya Protocol) applies in scope to material genetic resources and not to genomic sequence information per se, so it does not require benefit-sharing from users of genomic sequence data alone.

The terms of access and benefit-sharing under the Treaty are currently under review with the objective of increasing the flow of payments from users to the BSF, and possibly increase the scope of materials in the multilateral system. The review process was launched because, at the time, no mandatory payments have been made by commercial users. Very recently, the BSF received its first payment pursuant to the mandatory benefit-sharing conditions. Generally speaking, it appears that those users who might be obliged to make payments—for example, those whose product development and delivery strategy includes patenting new PGRFA products—have chosen not to obtain materials from the multilateral system. Some delegations have stated they will only endorse a revised multilateral system if it includes benefit-sharing from commercial users of genomic sequence information.

One option that is being considered is to create a subscription system, whereby PGRFA users could choose to subscribe to the multilateral system for 10 years, during which they would make annual payments to the BSF based on all their seed sales, regardless of whether or not all of their commercialized PGRFA products actually incorporate materials from the multilateral system of access and benefit-sharing. The subscription system could be the key to resolving tensions concerning genomic data. Payments from subscribers could be construed to reflect the commercial value of their own use of both genetic material and sequence data. In this way, the governance of both, both genetic materials and genomic data could be addressed under the same policy framework. Of course, all regions will need to agree on the rate of payments.

Another option that seems rational and straightforward from the point of view of research and development organizations conserving, accessing, distributing, and using genetic resources—but which is not currently getting much support from a number of contracting parties—would be for contracting parties to agree to make payments themselves to the ITPGRA benefit-sharing fund, based on total seed sales within their jurisdictions. In return, all users in those countries would enjoy facilitated access to genetic resources in the multilateral system and related genomic information. In this scenario, similar to the subscription option described earlier, the distinction between access and use of material genetic resources and genetic sequence data disappears for practical purposes, as do challenges linked to attribution, because payments to the benefit-sharing fund would presumably reflect the value of access to the genetic resource or gene sequence data, or both. It is hoped that these kinds of simplified approaches can help avoid disincentives rooted in unresolved controversies over benefit-sharing, and instead encourage proactive use of new-generation technologies in sustainable, equitable agricultural research and development.

## Notes

^a^ https://www.genesys-pgr.org/

^b^ https://ssl.fao.org/glis

^c^ https://www.cbd.int/abs/dsi-gr.shtml

^d^ http://snp-seek.irri.org/_snp.zul

^e^ https://www.crop-diversity.org/mgis/gigwa

^f^ https://sweetpotatogenomics.cals.ncsu.edu/

^g^ http://seedsofdiscovery.org/
